# Device-based measures of physical activity are associated with cognitive function but do not inoculate against later cognitive decline

**DOI:** 10.21203/rs.3.rs-10975193/v1

**Published:** 2026-09-30

**Authors:** Robert Eves, Justin Hachenberger, Fabio Franzese, Claudia Voelcker-Rehage, Angelina Sutin, David Richter, Sakari Lemola

**Affiliations:** Bielefeld University; Bielefeld University; SHARE Berlin Institute; University of Münster; Florida State University College of Medicine; SHARE Berlin Institute; Bielefeld University

**Keywords:** cognition, cognitive decline, accelerometery, physical activity, sleep, ageing

## Abstract

Physical activity and sleep are thought to be key protective factors against cognitive decline. However, longitudinal studies with objective measures of physical activity and sleep are still missing. 719 older adults from the Survey of Health, Ageing and Retirement in Europe (SHARE) wore accelerometers over an 8-day period. Cognitive function was measured at baseline and 26 months later. In unadjusted analyses, steps per day and intensity minutes per day at baseline were associated both contemporaneously and longitudinally with cognitive function (betas = 0.22 and 0.23, both *p* < 0.001). However, in longitudinal analyses that controlled for age, education, and cognitive function at the prior wave, physical activity was no longer significantly associated with cognitive function (beta = 0.04, *p* = 0.131). Sleep metrics of sleep duration and sleep efficiency were not significantly associated with cognitive function (all betas < 0.21, all *p values* > 0.090). Alongside other interpretations such as reverse causality, the results are consistent with a concurrent effect of physical activity but not with inoculation effects against cognitive decline 26 months later.

## Social media post of about 200 characters

In a study from 10 European countries, device-based metrics of physical activity but not sleep are concomitantly associated with cognitive performance in older adults.

## Introduction

Dementia and Alzheimer’s disease are among the leading causes of disability in the world (Wimo et al. 2017) and it is paramount to identify ways to decrease or delay cognitive decline (Livingston et al. 2020). Potential targets that might contribute to cognitive decline are physical activity (PA) levels and sleep (Phillips 2017; World Health Organization 2019; Sewell et al. 2021; Bloomberg et al. 2023). Both low levels of PA and poor sleep are associated with greater levels of dementia biomarkers and risk of onset of Alzheimer’s disease in older individuals (Bubu et al. 2016; Guure et al. 2017; Brown et al. 2019; Sabia et al. 2021; Iso-Markku et al. 2024).

Recently, studies have shown that PA may interact with sleep duration to predict trajectories of cognitive aging (Sewell et al. 2021). A review of cross-sectional studies and experimental studies without longer-term follow-up suggested that high levels of PA may buffer the potentially negative influence of poor sleep, including sleep duration that is too short or too long or sleep disturbances (Sewell et al. 2021). Furthermore, a recent longitudinal study with a follow-up over 10 years investigated the joint effects of self-reported physical activity and sleep duration on cognitive functioning in a sample of adults aged 50–95 years (Bloomberg et al. 2023). Instead of higher levels of PA buffering against the negative effects of poor sleep, the study found that more physically active older individuals with shorter sleep duration had the greatest cognitive decline. This meant their initial higher cognitive scores were not fully maintained in the long-term (Bloomberg et al. 2023). Moreover, sleep was a more important factor for cognitive functioning in the oldest individuals, indicating a further potential moderation effect by age. In addition to moderation (e.g., moderation of the effect of sleep duration on cognitive functioning by PA, implying a statistical interaction), sleep has also been suggested as a mediator of effects of PA. As there is evidence that PA is positively associated with numerous markers of sleep (Kredlow et al. 2015), improvements in sleep duration and efficiency may be a mechanism by which PA contributes to better cognitive functioning (Sewell et al. 2021). Based on past research, it is therefore currently unclear whether potential effects of PA on cognitive functioning could be moderated and/or mediated by sleep duration and efficiency, especially in longitudinal studies of older individuals.

A limitation of existing research on the joint role of PA and sleep for cognitive functioning and decline is that they largely rely on self-reported data and only focus on sleep duration and neglect other important sleep metrics, such as sleep efficiency (Gruber et al. 2014). Self-reports are prone to reporting-errors due to recall bias as well as common method bias and may not reflect PA and sleep accurately (Jackson et al. 2018; Podsakoff et al. 2024; Akre et al. 2025). Studies that have used actigraphy-assessment of PA and sleep have found contradictory findings, especially regarding interaction effects between PA and sleep patterns (Lambiase et al. 2014; Falck et al. 2018). This may be partially due to limited statistical power, as interactions need more statistical power than main effects. Moreover, other studies have focused on the association between physical exercise and cognitive functioning, rather than PA. Physical exercise is a special case of PA that involves intentional, planned, and structured PA (Caspersen et al. 1985) often at a higher intensity. An exclusive focus on physical exercise may overlook other types of incidental PA observed during the day, such as going for a walk, commuting, cleaning, gardening, or completing other daily chores. In addition, with one exception (Bloomberg et al. 2023), research on joint association of PA and sleep on cognitive functioning has been cross-sectional. To study not only cognitive performance, but also cognitive decline, it is necessary to use longitudinal designs in which PA and sleep are used to predict not only the concurrent levels of cognitive functioning but also its change over time.

The current study aims to examine cognitive functioning, and in particular longitudinal changes in cognitive functioning, in a sample of older adults. We used longitudinal data of participants aged between 50 and 96 years from the Survey of Health, Ageing and Retirement in Europe (SHARE) panel study in which both PA levels and sleep patterns were assessed by actigraphy over 8 days or more. Our primary aim was to determine whether device-measured physical activity (steps per day and intensity minutes) and sleep metrics (sleep duration and sleep efficiency) are associated with concurrent cognitive functioning, cognitive functioning assessed approximately 26 months later, and the relative change in cognitive functioning between the two timepoints. It is hypothesised that relatively long and short sleep durations is associated with lower cognitive functioning at baseline, after 26 months, and the relative change in cognitive functioning between the two timepoints. In contrast to the potential non-linear effect of sleep duration, we hypothesise positive linear relationships for sleep efficiency and metrics of PA for cognitive functioning. As a secondary aim, we test for moderation between metrics of PA and sleep to test the hypothesis that high levels of PA may offset the potentially negative association of poor sleep. Moreover, as past research indicates these associations and interaction patterns might become greater as participants get older, age will be tested as an additional moderator of effects. Finally, given the potential role of PA on sleep, we test the alternative hypothesis that the association between PA and cognitive functioning is mediated by sleep.

## Methods

### Data sources

SHARE is a cross-national panel survey of European adults aged 50 and older. SHARE was established in 2004 with representative data from each participating country collected every two years using face-to-face interviews (Börsch-Supan et al. 2013). For this analysis, a subset of the data from the 8th and 9th wave of data is used, collected between 2019 and 2020 and between 2021 and 2022, respectively (Bergmann et al. 2024a, b). The SHARE study is subject to continuous ethics review with waves eight and nine reviewed and approved by the Ethics Council of the Max Planck Society. In addition, the country implementations of SHARE were reviewed and approved by the respective ethics committees or institutional review boards as required. All participants provided informed consent.

#### SHARE Accelerometer Study- Accelerometer data collection (physical activity and sleep data)

As part of the 8th wave of data collection, the SHARE Accelerometer Study was conducted in a subset of ten participating countries, ensuring geographic variation: Belgium, Czech Republic, Denmark, France, Germany, Italy, Poland, Slovenia, Spain, and Sweden (Bergmann et al. 2024b; Franzese and Benjamin 2025). A target sample of 200 participants per country was based on each country’s original longitudinal sample, stratified by age group and activity level that was self-reported in the previous wave (Cohn-Schwartz and Khalaila 2022). A total of 4330 participants were contacted for participation in the SHARE Accelerometer Study; the consent rate was 54.4%. Due to suspension of fieldwork caused by the COVID pandemic, however, the final subsample had 855 participants with valid accelerometer measurements (Franzese and Benjamin 2025).

In the SHARE Accelerometer Study, participants were asked to continuously wear an accelerometer (Axivity AX3, Axivity Ltd, UK) for eight consecutive days. They were instructed to wear the accelerometer on their upper thigh, attaching it using adhesive tape. The details of the data processing of the raw accelerometer data that has been previously published (Bergmann et al. 2024b). Briefly, the data was processed using the ActiPASS Version 1.61 beta software (Hettiarachchi and Johansson 2025). ActiPASS derives activities and postures, such as standing, sitting, walking, and cycling, based on thigh-worn actigraphy data (Skotte et al. 2014), and has been subsequently validated (Johansson et al. 2023). Two PA metrics and two sleep metrics derived from ActiPASS were subsequently used in this analysis. For PA, these were the number of steps per day and the number of minutes spent doing intense activities per day. For the metric of intensity minutes, the following activities were considered: fast walking (i.e., cadence ≥ 100 steps/min), running, cycling, stair climbing, and other periodic movements with a cadence ≥ 100 steps/min (Bergmann et al. 2024b; Franzese and Benjamin 2025). Based on how many observation days there were for each participant, the average number of steps and number of minutes spent doing intense activities per day were calculated and then standardised, with original scores of 0 deemed implausible and thus removed. Sleep metrics were also averaged per day, with the prerequisite that a participant had at least 3 nights of valid data. In order to test for non-linear effects, sleep duration, originally measured in minutes, was z standardised and then categorised into 3 groups of low (Z <−1, equivalent to less than 5 hours and 48 minutes of sleep), medium (−1 < Z < 1) and high (Z > 1, equivalent to more than 8 hours and 45 minutes of sleep). This is broadly comparable to previously used categories of too little and too much sleep of less than 6 hours and more than 9 hours, respectively (Shah et al. 2025). Sleep efficiency, calculated by dividing the time spent asleep versus the time spent in bed, was maintained as a continuous variable but was standardised for all statistical analyses. After z scores for the sleep and PA were created, five participants were removed due to implausible or extreme outlier values (z scores < – 4 or > 4).

#### Cognitive Functioning

As part of the main SHARE study, cognitive functioning was assessed at both Waves 8 and 9 by combining scores on three cognitive tasks: immediate recall, delayed recall, and verbal fluency. In the immediate recall task, the interviewer read aloud ten words and asked the participant to repeat as many words as possible. For the delayed recall task, the participant was asked to repeat these words again, approximately 5 minutes later. The scores for these two tasks ranged from 0 to 10 points. Verbal fluency was measured by asking participants to name as many animals as possible in 60 seconds. The average of the standardized scores on these three measures at each wave was used as the measure of cognitive functioning. A previous factor analysis of these tasks confirmed the single underlying measure (Cohn-Schwartz and Khalaila 2022).

#### Covariates

Covariates were age (in years), gender, education, and financial distress. Based on the original ISCED-1997 classification of education, which includes 7 education levels from 0 = none to 6 = second stage of tertiary education, education was categorised into low (0–2), medium (3–4) and high (5–6) (UNESCO United Nations Educational 2003). Financial distress was based on the question “Thinking of your household’s total monthly income, would you say that your household is able to make ends meet?” which was coded with a minimum score of 0 meaning “with great difficulty” while a maximum score of 3 indicated “easily”. When participants were not directly asked this item, the response of their partner in the household was used instead.

### Statistical analysis

Participants with complete data for measures of PA at wave 8, sleep at wave 8, and cognitive functioning at both Waves 8 and 9 were included for analysis. Missing covariate data was imputed using MICE, resulting in 0.62% of covariate data being imputed. Initially, four unadjusted cross-sectional regression analyses tested each unique combination of sleep metrics (sleep duration, efficiency) and PA metrics (steps, intensity minute) for predicting cognitive functioning at wave 8 (Cross Sectional Models A-D). Due to the similarity of the cross-sectional results, unadjusted longitudinal analyses tested only sleep efficiency and PA intensity at Wave 8 as predictors of cognitive functioning at Wave 9 (Longitudinal Model A). Age, gender, education and financial distress were then added as covariates (Longitudinal Model B). Finally, cognitive functioning at Wave 8 was added as an additional predictor of cognitive functioning at Wave 9 (Longitudinal Model C). This addition was to test whether the sleep and PA metrics explained additional variance in cognitive functioning after controlling for cognitive functioning 2 to 3 years prior, thus testing for prediction in change in cognitive function over time. To test the hypothesis that high PA may buffer the risk from poor sleep, we included two-way interactions between the sleep and PA metrics, as well as their main effects. Alternatively, to test the theory that sleep metrics mediate the association between PA and cognitive functioning, we performed two mediation analyses. The first mediation analysis was unadjusted and only included PA and sleep metrics at Wave 8 as predictors of cognitive functioning at Wave 9. The second mediation analysis was adjusted with the covariates and cognitive functioning at Wave 8. All analyses were performed in R. The mediation analysis used the package Psych (Revelle 2020), which uses bootstrapped confidence intervals for the indirect effects. All multivariable regression were 2-sided with P < .05 indicating statistical significance. The preregistration for these specific analyses can be found on the OSF- https://osf.io/fqkbd. Following the completion of our pre-registered analyses, we then completed a number of post-hoc analyses as tests for robustness. These included using sex specific sleep z scores, as there are known sex differences in sleep duration (van den Berg et al. 2009), and testing by cognitive subtest, as to identify differences in domains related to memory and verbal fluency.

## Results

The analytic sample included 719 participants with valid actigraphy data at Wave 8 and valid cognitive function scores at Waves 8 and 9. On average, raw scores on the cognitive tasks slightly declined between Wave 8 and 9 (Table 1), equivalent to an overall reduction in cognitive scores of 0.12 SD (1.8 IQ points). Descriptive statistics for all variables are in Table 1, while the correlation matrix of continuous variables is shown in Table S1. Compared to the main SHARE study, participants in the accelerometer study were slightly more likely to be younger; there were no significant differences by gender or education, see Tables S2 and S3.

Table 1: **Characteristics of the analytic sample (n = 719)**

### Cross Sectional Analyses- Sleep, PA and Cognitive Functioning at Wave 8

Four multiple regressions were performed to examine the association between the PA metrics, sleep metrics and cognitive function (Table 2 and Fig. 1). In separate analyses, as expected, the number of steps per day and the number of intensity minutes were significantly and similarly associated with better cognitive functioning (all betas > 0.20, both *p* < 0.001), see Table 2.

In contrast to the PA metrics, there were no significant linear or non-linear relationships between the sleep metrics and cognitive functioning (Table 2 and Fig. 1). In comparison to those with a medium level of sleep duration, having long sleep duration (i.e. 8 hours and 45 minutes or more) (beta = −0.20, *p* = 0.090) or short sleep duration (i.e. 5 hours and 48 minutes or less) (beta = −0.08, *p* = 0.469) was not associated significantly with cognitive functioning (Table 2, Cross Sectional Models A and D). Similarly, sleep efficiency was not significantly associated with cognitive functioning (beta = 0.05, *p* = 0.186), see Table 2, Cross Sectional Models B and C. Across all four regressions, there was no supporting evidence that higher levels of PA buffered potentially detrimental effects of short or long sleep duration or poor sleep efficiency (all interactions *ns*, Table 2, Cross Sectional Models C and D)).

#### Longitudinal Analyses- Sleep, PA and Cognitive Functioning at Wave 8 Predicting Cognitive Functioning at Wave 9

Due to the similarity in results from the different PA and sleep metrics, and the high correlation between the two indices of PA (number of steps and intensity minutes, r = .93) and between the two sleep indicators (sleep efficiency and sleep duration in minutes, r = 0.41) (Table S1), we used intensity minutes and sleep efficiency in the main longitudinal analyses, with models using number of steps per day and sleep duration reported in the supplementary material, see Table S4.

Similar to the analyses using cognitive functioning data at Wave 8, the intensity minutes at Wave 8 were associated with cognitive function at Wave 9 (beta = 0.27, *p* < 0.001), see Table 3, Longitudinal Model A. There was no significant association between sleep efficiency at wave 8 and cognitive functioning at Wave 9, nor a significant moderation between sleep efficiency and intensity minutes (−0.01, p = 0.718), see Table 3, Longitudinal Model A. The addition of covariates indicated that younger age, female gender, lower financial distress and higher education levels were associated with better cognitive function (Table 3, Longitudinal Model B) and partially confounded the association between PA intensity minutes and cognitive function at Wave 9 (beta 0.11, *p* = 0.004). Age’s association with cognitive performance appeared to be independent of sleep and PA metrics, as indicated by the lack of significant interaction effects, see Figure S1. In Longitudinal Model C, cognitive function at Wave 8 was the strongest predictor of cognitive function at Wave 9 (beta = 0.64, *p* < 0.001) and attenuated the association between intensity minutes and cognitive function at Wave 9 to non-significance (beta = 0.04, *p* = 0.131).

In order to examine whether sleep efficiency at Wave 8 mediated the association between intensity minutes at Wave 8 and cognitive function at Wave 9, we performed an unadjusted and adjusted mediation analysis (Fig. 2). In the unadjusted model, PA intensity minutes significantly predicted cognitive function (c = 0.27, *p* < 0.001) and remained a significant predictor after sleep efficiency was added as a mediator (c’ = 0.26, *p* < 0.001). In the unadjusted model intensity minutes also significantly predicted sleep efficiency (a = 0.22, *p* < 0.001) but sleep efficiency did not significantly predict cognitive function when controlling for intensity minutes (b = 0.03, *p* = 0.479). In the adjusted model, when covariates and cognitive functioning at Wave 8 were included, the association between intensity minutes and cognitive function at Wave 9 was no longer significant (c’ = 0.05, p = 0.101), see Fig. 2. Similar results were found when instead using sleep duration and steps per day (Table S5). Overall, in both the unadjusted and adjusted model, sleep metrics did not show any mediating effect on the relationship between PA intensity minutes and cognitive functioning at Wave 9.

### Post Hoc Tests for Robustness

As exploratory analyses, we re-standardised the sleep metrics separately for males and females and then reperformed the cross-sectional analyses. In the model including intensity minutes and sleep duration, the unadjusted association between high levels of sleep and cognition was found to be statistically significant (beta = −0.23, *p* = 0.038), see Table S6. In contrast the sleep efficiency metric continued to not be significantly associated with cognition at wave 8 (beta = 0.03, p = 0.470).

Instead of using total cognitive scores, the second robustness tested the cognitive subdomains separately (Table S7). Regardless of whether the cognitive test measured immediate recall, delayed recall, or verbal fluency, there were consistent significant associations between physical activity and cognitive performance in both cross-sectional and longitudinal analyses, which did not adjust for baseline cognition, with beta values ranging from 0.15 to 0.23 (all p < 0.001).

## Discussion

The current study shows that device-measured PA is cross-sectionally and longitudinally associated with cognitive function. In unadjusted analyses, an increase of 1 SD in intensity minutes per day is associated with an increase of approximately 0.22 and 0.26 SD in cognitive function in cross-sectional and longitudinal analyses, respectively. However, PA did not longitudinally predict cognitive function 26 months later when controlling for baseline cognitive function. According to our pre-registered analyses, device-measured sleep duration and efficiency were not associated with cognitive function in cross-sectional analyses nor in longitudinal analyses. There was also no evidence for moderation or mediation of the association between PA and cognitive function by actigraphy sleep indices, nor was there evidence of interaction effects by age.

The unadjusted significant associations between actigraphy PA and cognitive function is consistent with evidence from previous studies that used actigraphy PA (Lambiase et al. 2014; Falck et al. 2018; Wilckens et al. 2018) or self-report PA (Guure et al. 2017; Phillips 2017; Sewell et al. 2021; Bloomberg et al. 2023); for a recent meta-analysis see Iso-Markku et al., 2024 (Iso-Markku et al. 2024). The mechanisms behind these associations may involve cellular and molecular changes triggered by physical activity such as changes in neurotrophic factors (Huang et al. 2014), neurogenesis (Yau et al. 2014), inflammation, angiogenesis (Schmidt et al. 2013), hypothalamus-pituitary-adrenocortical axis activity (Stillman et al. 2016), and autonomic function (Kashihara et al. 2009), which may mediate changes in brain structure and function (Stillman et al. 2016). This may be particularly important when one considers why we did not observe a significant longitudinal association after controlling for cognition at the prior wave. It is conceivable that the association between PA and cognitive function may be restricted to relatively acute effects that largely dissipate soon after the high levels of PA are discontinued. Thus, our results indicate that PA may concurrently improve cognition but cannot “inoculate” against cognitive decline 26 months later. This finding is in contrast though with evidence indicating that higher levels of self-reported PA at baseline predicts reduced decline in episodic memory recall up to 15 years later (Stenling et al. 2022). Overall, while the unadjusted association between PA and cognition appears robust, it is currently unclear whether the association of PA and cognitive decline is dependent on how PA is measured or the length of follow up.

Rather than PA having a unidirectional effect on cognition, there are two other possible alternative interpretations. First, the inverse direction of causality is possible whereby individuals with cognitive decline may be less willing or able to live an active life (Daly et al. 2015). Second, it is possible that there is a non-causal association between the two variables which could be due to a causal factor common to both PA and cognitive function, such as genetic variants. In order to estimate the strength of all possible causal paths, future studies should examine how contingent change in cognitive function is to changes in PA in a more fine-grained manner by having repeated measures of cognition and actigraphy based measured of PA. Such analyses could make use of panel data and apply statistical models such as the dual change score model (Mund and Nestler 2019) to distinguish acute and reversible effects of PA from those that are long-term and biologically ingrained.

The marginal non-linear association between actigraphy sleep duration and cognitive function was consistent with a recent systematic review and one meta-analysis indicating a relatively weak u-shaped association of device-measured sleep duration and subsequent cognitive decline (Carpi et al. 2024; Yang et al. 2024). Several mechanisms have been suggested to explain the association of short and long sleep duration with cognitive decline including increased inflammation leading to neurodegeneration (Patel et al. 2009; Lin et al. 2018) and increase amyloid plaques deposition (Ooms et al. 2014). Our finding of no significant association between actigraphy sleep efficiency and subsequent cognitive decline is inconsistent with the evidence summarized by Carpi et al. (2024) and could be due to the placement of the device on the thigh in the current study for which the measurement of sleep efficiency is not validated (Johansson et al. 2023; Carpi et al. 2024). Relatedly, actigraphy sleep duration and sleep efficiency did not moderate the association between actigraphy PA and cognitive function at baseline or follow-up.

The present study has several strengths. To our knowledge it is the first to address whether device-based measures of sleep moderate the longitudinal association between device-based measures of PA and cognitive function. The use of device-based measures for both PA and sleep has the advantage that it is not confounded by reporting bias and common method bias as both self-reported PA and sleep may for instance be confounded by depressive symptoms (Podsakoff et al. 2024; Collins et al. 2025). Further strengths include the longitudinal design and the use of samples from several European countries. At the same time the study is not without limitations. First, the follow-up period was only 26 months and it is possible that findings would be different over a longer follow-up period as on average cognitive function decreased only by 0.13 SD over this follow-up period. Second, while the sample was quite large for a study using device-based measures of PA and sleep, the statistical power to detect small effects and interaction effects in particular was limited. However, simulation studies suggest a sample of 500 to be sufficient when an interaction effect size is moderate (*R* ^2^ = .20) but not when it is small (*R* ^2^ = .10) (Jolicoeur-Martineau et al. 2020). Thus, our ability to detect a small effect of sleep on cognitive function, and especially two- and three-way interactions involving PA, sleep and age, may have been limited. Third, the measurement of PA and sleep with actigraphy on the middle of the thigh is not as well validated as at other wear positions and therefore it was not possible to distinguish between different intensities of physical activity. Relatedly, the metric of device-measured sleep efficiency may involve a larger amount of unsystematic variance compared to wrist-worn actigraphy.

## Conclusions

The present study shows an unadjusted cross-sectional and longitudinal association between actigraphy PA and cognitive function. Actigraphy PA, however, was unrelated to changes in cognitive function over 26 months when age, sex, education, and baseline cognitive function were controlled for. Thus, while analyses are correlational in nature, results are consistent with a concurrent effect of PA but not with inoculation effects against cognitive decline until 26 months later. Device-based sleep duration and sleep efficiency metrics were not significantly related to cognitive function nor was there evidence that sleep moderated or mediated the association between PA and cognitive function. Overall, future cohort studies should analyse correlated change to determine whether changes in PA concur with changes in cognitive function.

## Supplementary Material

Supplementary Files

This is a list of supplementary files associated with this preprint. Click to download.

- SupMaterial.docx

## Figures and Tables

**Figure 1:**
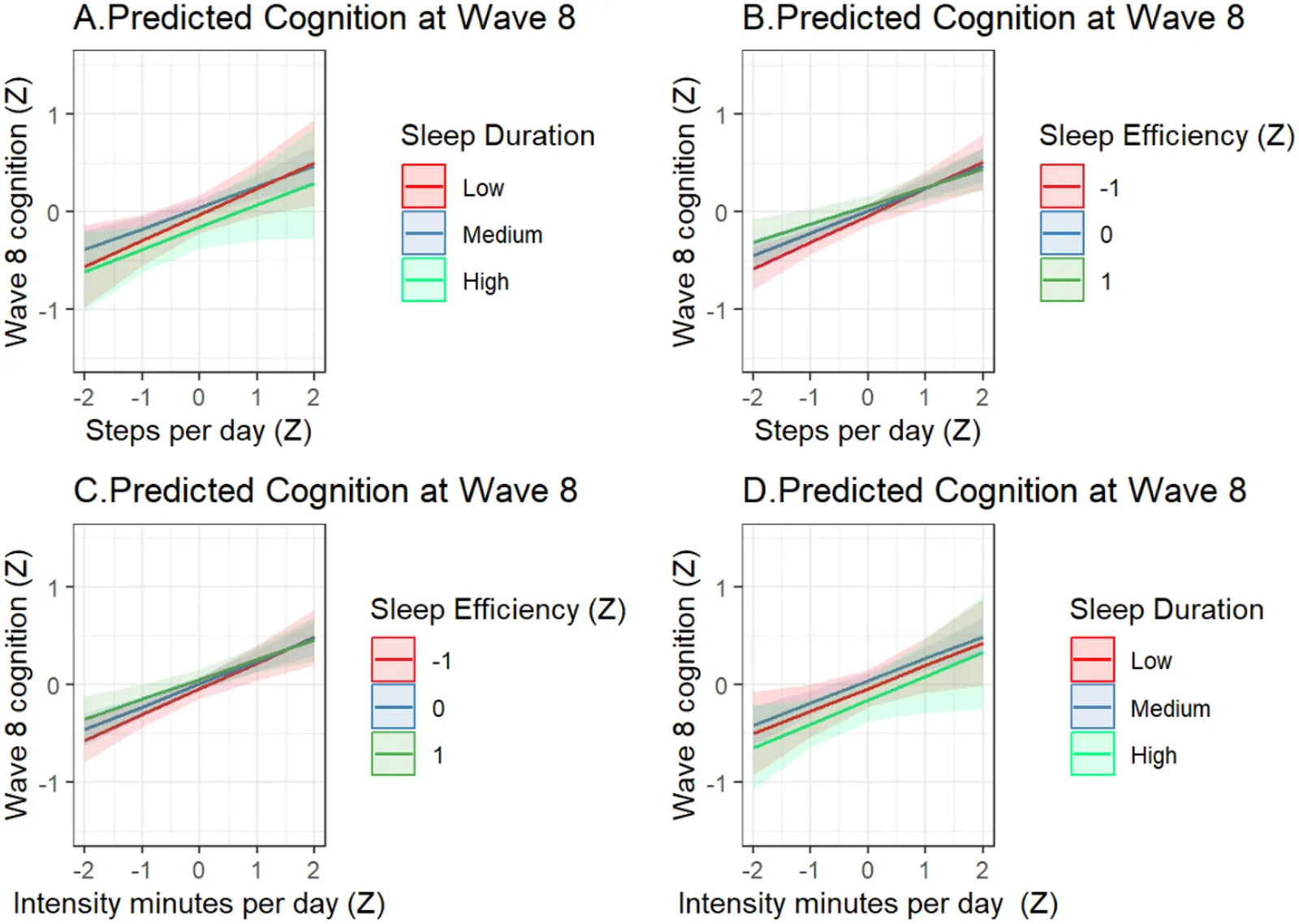
Unadjusted, cross-sectional associations between sleep metrics, activity metrics, and cognitive function at Wave 8

**Figure 2:**
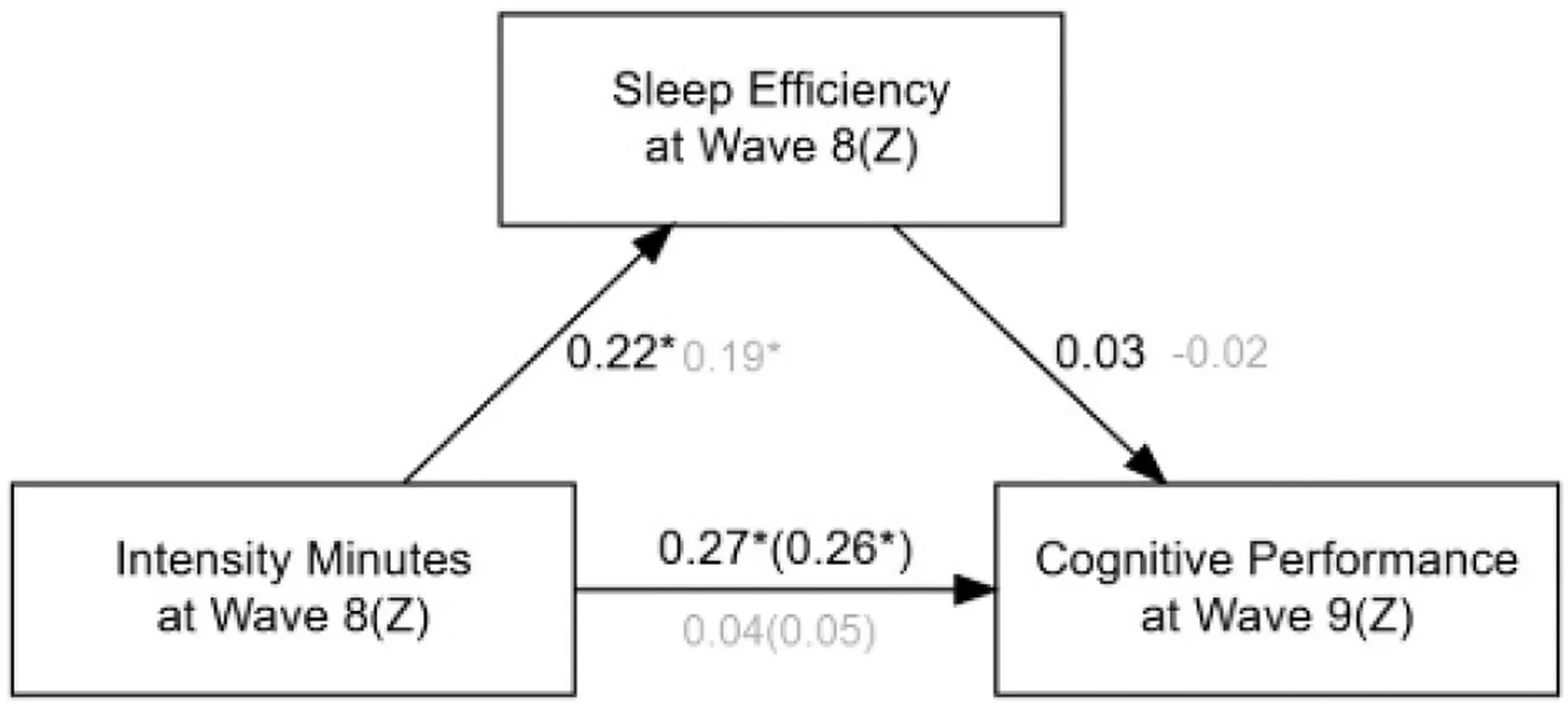
Mediation Analysis. Parameters from the unadjusted analysis (black) and adjusted analysis (grey), determining whether sleep efficiency (Wave 8) mediated the association between the device-measured PA metric of intensity minutes (Wave 8) and cognitive function at Wave 9, before and after adjusting for covariates (gender, age, education levels, and financial distress) and cognitive function at Wave 8

**Table 1:** Overview of key variables

Actigraphy- Steps Per Day	Mean	SD	Min	Median	Max
	7597.1	3651.3	1.0	7286.4	22097.4
Actigraphy- Steps Per Day (Z)	−0.0	1.0	−2.0	−0.1	3.8
Actigraphy- Intensity Minutes Per Day	53.3	28.5	0.0	49.5	160.6
Actigraphy- Intensity Minutes Per Day (Z)	−0.0	0.9	−1.8	−0.1	3.5
Actigraphy- number of nights of sleep data available	7.5	1.1	3.0	8.0	10.0
Actigraphy- Sleep Duration (hours)	7.3	1.5	2.8	7.3	14.1
Actigraphy- Standardised Sleep Duration (Z)	0.0	1.0	−3.0	−0.0	4.5
Actigraphy- Sleep Efficiency (%)	84.0	9.0	48.6	85.7	100.0
Actigraphy- Standardised Sleep Efficiency (Z)	−0.0	1.0	−4.0	0.2	1.8
Wave 8- Verbal fluency score (number of animals)	22.1	7.1	2.0	22.0	54.0
Wave 9- Verbal fluency score (number of animals)	21.7	7.4	1.0	21.0	60.0
Wave 8 delayed recall (number of words)	4.2	2.1	0.0	4.0	10.0
Wave 9 delayed recall (number of words)	4.0	2.1	0.0	4.0	10.0
Wave 8 immediate recall (number of words)	5.6	1.6	0.0	6.0	10.0
Wave 9 immediate recall (number of words)	5.4	1.7	0.0	5.0	10.0
Wave 8 Cognitive Performance (IQ Points)	100.0	15.0	53.5	100.3	139.3
Wave 9 Cognitive Performance- standardised on Wave 8 (IQ Points)	98.2	15.5	49.3	98.7	142.0
Household Financial Distress (only self-report)	1.9	0.9	0.0	2.0	3.0
Household Financial Distress (self or partner report)	2.0	0.9	0.0	2.0	3.0
Age of respondent at the time of interview	70.5	8.7	52.0	70.0	96.0
		N	%	
Actigraphy- Sleep Duration Categorised	Medium	521	72.3		
	Low (< 5.81 hours)	103	14.3	
	High (> 8.76 hours)	97	13.5	
Participant Sex	male	293	40.6		
	female	428	59.4	
Wave 8 ISCED Education Level	low	221	30.7		
	medium	321	44.5	
	high	175	24.3	

**Table 2:** Cross Sectional Analyses- Wave 8 Cognitive Scores as the Outcome

*Predictors*	Cognitive function Wave 8			Cognitive function Wave 8			Cognitive function Wave 8			Cognitive function Wave 8		
	*Estimates*	*CI*	*p*	*Estimates*	*CI*	*p*	*Estimates*	*CI*	*p*	*Estimates*	*CI*	*p*
(Intercept)	0.04	−0.04–0.12	0.346	0.01	−0.06–0.08	0.758	0.01	−0.06–0.08	0.791	0.04	−0.04–0.13	0.327
Actigraphy-Sleep Duration Categorised: Low (ref: Medium)	−0.07	−0.28–0.14	0.515							−0.08	−0.28–0.13	0.469
Actigraphy-Sleep Duration Categorised: High (ref: Medium)	−0.20	−0.43–0.03	0.090							−0.20	−0.43–0.03	0.090
Actigraphy-Steps Per Day (Z)	0.22	0.13–0.30	**< 0.001**	0.23	0.15–0.31	**< 0.001**						
Sleep Duration Categorised: Low X Steps Per Day (Z)	0.05	−0.16–0.26	0.649									
Sleep Duration Categorised: High X Steps Per Day (Z)	0.01	−0.23–0.25	0.926									
Actigraphy-Standardised				0.05	−0.02–0.12	0.186	0.05	−0.03–0.12	0.195			
Sleep Efficiency (Z)												
Sleep Efficiency (Z) X Steps Per Day (Z)				−0.04	−0.12–0.03	0.281						
Actigraphy-Intensity Minutes Per Day (Z)							0.23	0.16–0.31	**< 0.001**	0.23	0.14–0.32	**< 0.001**
Sleep Efficiency (Z) X Intensity Minutes Per Day (Z)							−0.03	−0.11–0.05	0.456			
Sleep Duration Categorised: Low										0.01	−0.21–0.22	0.958
X Intensity Minutes Per Day (Z)												
Sleep Duration Categorised: High X Intensity Minutes Per Day (Z)										0.02	−0.23–0.27	0.887
R^2^ / R^2^ adjusted	0.055 / 0.049			0.055 / 0.051			0.056 / 0.052			0.57 0.050		

**Table 3:** Longitudinal Analyses- Wave 9 Cognitive Scores as the Outcome

*Predictors*	A. Cognitive function Wave 9			B. Cognitive function Wave 9			C. Cognitive function Wave 9		
	*Estimates*	*CI*	*p*	*Estimates*	*CI*	*p*	*Estimates*	*CI*	*p*
(Intercept)	0.01	−0.07–0.08	0.842	−0.76	−1.00 – −0.52	**< 0.001**	−0.10	−0.29–0.09	0.294
Sleep Efficiency (Z)	0.02	−0.05–0.10	0.534	−0.02	−0.09–0.05	0.592	−0.02	−0.07–0.03	0.393
Intensity Minutes (Z)	0.27	0.19–0.34	**< 0.001**	0.11	0.04–0.18	**0.004**	0.04	−0.01–0.10	0.131
Sleep Efficiency (Z) x Intensity Minutes (Z)	−0.01	−0.09–0.06	0.718	−0.02	−0.09–0.06	0.644	−0.00	−0.06–0.05	0.867
Age (years)				−0.04	−0.05 – −0.03	**< 0.001**	−0.02	−0.03 – −0.02	**< 0.001**
Female Gender (reference: Male)				0.15	0.02–0.28	**0.021**	−0.01	−0.11–0.09	0.911
Education Level - Medium (reference: Low)				0.41	0.26–0.56	**< 0.001**	0.14	0.02–0.25	**0.018**
Education Level - High (reference: Low)				0.71	0.54–0.89	**<0.001**	0.23	0.09–0.37	**0.001**
Financial Distress				0.11	0.04–0.18	**0.002**	−0.01	−0.06–0.05	0.823
Sleep Efficiency (Z) x Age (years)				−0.00	−0.01–0.00	0.286	−0.00	−0.01–0.00	0.406
Intensity Minutes (Z) x Age (years)				0.01	−0.00–0.01	0.217	−0.00	−0.01–0.00	0.657
Sleep Efficiency (Z) x Intensity Minutes (Z) x Age (years)				−0.00	−0.01–0.01	0.795	−0.00	−0.01–0.00	0.681
Cognitive function at Wave 8 (Z)							0.64	0.59–0.70	**< 0.001**
R^2^ / R^2^ adjusted	0.066 / 0.062			0.297 / 0.286			0.597 / 0.591		

## Data Availability

The SHARE data can be freely accessed after completion of a registration. More information can be found here: https://share-eric.eu/data/data-access.

## Code Availability

The preregistration and R script used for this specific analysis can be found here: https://osf.io/fqkbd
